# A Case of Thyroid Storm Associated with Cardiomyopathy and Poststreptococcal Glomerulonephritis

**DOI:** 10.1155/2016/7192359

**Published:** 2016-10-27

**Authors:** Lisa J. Underland, Gerson A. Vallencia Villeda, Abhijeet Pal, Leslie Lam

**Affiliations:** ^1^Department of Pediatric Endocrinology and Diabetes and Department of Pediatric Nephrology, The Children's Hospital at Montefiore, 3415 Bainbridge Ave., Bronx, NY 10467, USA; ^2^Department of Cardiology, Columbia University Medical Center, Room 255, 3959 Broadway, New York, NY 10032, USA

## Abstract

Thyroid storm has a high mortality rate and is often associated with a precipitating factor such as intercurrent illness or infection. It is rare in pediatric patients. Cardiac disease in hyperthyroidism mostly manifests itself as tachycardia but more serious cardiac findings have also been described. A 5-year-old male with recent strep throat infection presented with dilated cardiomyopathy, hematuria, and symptoms and lab findings consistent with severe hyperthyroidism. He was diagnosed with thyroid storm secondary to concurrent Graves' disease and poststreptococcal glomerulonephritis (PSGN). After starting the treatment with methimazole and a beta-blocker, his cardiac disease gradually improved and the PSGN resolved over time. There are no specific pediatric criteria for thyroid storm. Adult criteria can be difficult to apply to pediatric cases. Criteria for diagnosis of thyroid storm are less clear for pediatric patients. Dilated cardiomyopathy is a rare cardiac manifestation of hyperthyroidism. PSGN is due to glomerular immune complexes and can complicate group A strep infection. Providers should be aware of cardiac disease as a complication of hyperthyroidism. PSGN should not mechanistically be related to hyperthyroidism but can precipitate the signs of thyroid storm such as hypertension. This association has not been previously reported in the literature.

## 1. Introduction

Hyperthyroidism occurs with an incidence of 1 per 100,000 children, mostly commonly in postpubertal females [1]. Prepubertal patients represent a diagnostic challenge with more atypical signs and symptoms at presentation which may lead to a delay in diagnosis. Thyroid storm is an extreme form of hyperthyroidism with a more severe presentation that is associated with a high mortality rate [2]. It tends to occur in hyperthyroid patients with a precipitating factor such as surgery, infection, noncompliance with antithyroid medication, or radioactive iodine ablation. Whereas there are well-established clinical guidelines in adults for the diagnosis of thyroid storm, no such criteria yet exist for pediatric patients [2]. We describe an unusual case of thyroid storm in a young child with associated cardiomyopathy and poststreptococcal glomerulonephritis.

## 2. Case

A previously healthy 5-year-old African American male presented to the emergency department with a 9-day history of lethargy, fever, vomiting, weight loss, and diarrhea. The week before presentation, he was diagnosed with streptococcal pharyngitis via rapid antigen testing. He was prescribed a course of oral antibiotics but was noncompliant. On presentation, he was tall and thin (BMI < 3rd percentile, height > 95 percentile) with noticeable goiter and prominent proptosis. He was agitated, restless, tachycardic, and hypertensive (heart rate: 140 beats per minute, blood pressure: 154/99 mmHg). The mother noted that he had always been tall and thin. She had not noted any symptoms of hyperthyroidism such as weight loss, increased appetite, palpitation, or diarrhea prior to this illness. Chest X-ray showed cardiomegaly. Echocardiogram revealed dilated cardiomyopathy with severely dilated left ventricle with severely decreased left ventricular systolic function. Preliminary labs showed significantly elevated pro B-type natriuretic peptide at 19,632 (reference range is <450 pg/mL), suppressed TSH <0.005 *μ*U/mL, and elevated free T4 >7.77 ng/dL consistent with severe hyperthyroidism and cardiomyopathy. Based on his clinical presentation and his significant cardiac findings, he was diagnosed with thyroid storm and was admitted to the pediatric ICU for further monitoring and management.

In the PICU, he was started on atenolol 25 mg daily and methimazole 5 mg twice daily for hyperthyroidism and given a dose of penicillin G for treatment of his streptococcal pharyngitis. Streptozyme test was positive. He was also noted to have persistent microscopic hematuria and 30 mg/dL of protein on the urine dipstick. Pediatric nephrology was consulted. Renal workup revealed low C3 (60 mg/dL; reference range is 85–288) and normal C4 complement levels (30 mg/dL; reference range is 17–64). He had normal creatinine for his age (0.3 mg/dL). An ASO titer was elevated at 857 (reference range: 0–207 IU/mL). Given the history of recent streptococcal pharyngitis, elevated ASO and streptozyme levels, microscopic hematuria, and low C3, he was presumed to have poststreptococcal glomerulonephritis and was followed up conservatively. Because of the elevated thyroid stimulating immunoglobulin at 464%, he was diagnosed with Graves' disease. Over the next several days, his heart rate, blood pressure, and mental status improved and his thyroid function tests normalized (see Table 1). His left ventricular function also showed significant improvement after three weeks of treatment. He was discharged home on methimazole and atenolol on hospital day 15. Hematuria slowly resolved after discharge over the next few days. Repeat labs performed 8 weeks after hospitalization showed normal electrolytes and C3 levels, confirming the diagnosis of poststreptococcal glomerulonephritis. At follow-up, he had a bone age study done that showed significant advanced age (bone age of 11 years and 6 months at a chronological age of 5 years and 11 months) consistent with history of long-standing hyperthyroidism. Atenolol was discontinued several months later. He remained on methimazole at a maintenance dose.

## 3. Discussion

Thyroid storm in the setting of Grave's disease often presents after a precipitating factor. Our patient presented after an episode of acute streptococcal pharyngitis with cardiomyopathy. Thyroid storm is rare in both pediatric patients and adults. The diagnostic criteria are derived from adult thyroid guidelines, where several scoring systems exist. Historically, the most commonly used is the Burch-Wartofsky criteria, which attribute a severity score of 1–5 to individual hallmark features of the disease [2]. These include temperature, CNS effects, GI-hepatic dysfunction, cardiovascular dysfunction/heart failure, and precipitant history. Our patient had a score of 70, with ≥45 being considered highly suggestive and <25 being unlikely (25–44 supports the diagnosis).

In pediatric patients, the reliability and applicability of these scoring systems are less clear as compared with adults. With prior illness being a common trigger, the etiology of fever may be difficult to attribute to thyroid disease alone. Prepubertal patients in particular have higher resting heart rates at baseline than adults and adolescents. As such, a scoring system for thyroid storm that does not use pediatric age-specific standards for heart rate and temperature is problematic. In addition, CNS dysfunction in a child may be difficult to establish without accounting for developmental staging and age appropriate behavior.

A literature search of thyroid storm in the pediatric population reveals mostly case reports and small case series. Aslan et al. described a case of an 11-year-old patient who presented with a similar condition to our patient with fever, tachycardia, and hypertension in the setting of a viral illness [1]. This patient was diagnosed using the Burch-Wartofsky criteria with a score of 60. Lee and Hwang published a case series of pediatric patients with thyroid storm precipitating seizures [3]. While it is not clear what specific criteria were used to diagnose these patients, all of them had fever, tachycardia, and hypertension. Majlesi et al. described a case of thyroid storm caused by levothyroxine ingestion in a 2-year-old [4]. Further studies are needed to determine whether adult criteria for diagnosis can be adapted for use in kids although this may prove difficult given the low incidence of the disease in the pediatric population.

Poststreptococcal glomerulonephritis is an uncommon complication of group A streptococcal infections. The incidence is 1.7 to 13.2 per 100,000 and median age is 6–8 years [5]. The pathogenesis is incompletely understood, but it is known that the group A strep strain triggers a glomerular immune complex which leads to complement activation. This generally occurs at about 1–6 weeks from the initial infection. Symptoms can be mild with microscopic hematuria or severe with nephritic syndrome accentuated by gross hematuria, edema, hypertension, proteinuria, and elevated creatinine levels. Labs are characterized by low complement levels, specifically C3 and C50. These levels tend to decrease in 4–8 weeks. Diagnosis is based on clinical findings and documentation of recent group A strep infection (either a positive throat culture or positive streptococcal antibody tests such as a streptozyme test). Renal biopsy is not necessary unless there is uncertainty with the diagnosis. Treatment is supportive. For our patient, a presumptive diagnosis was made based on the findings on presentation which included recent streptococcal infection, hematuria, hypertension, and low C3 levels, although it is debatable whether the hypertension was a manifestation of hyperthyroidism or kidney disease. Further supporting the diagnosis in this patient is the self-limited course of his disease, the documentation of a prior group A streptococcal infection, and normalization of complement levels following disease resolution [5]. Dilated cardiomyopathy (DCM) is characterized by ventricular chamber enlargement and systolic dysfunction which presents as progressive heart failure. The annual incidence of DCM in children is 0.57 cases per 100,000 children and is the most common reason for cardiac transplant in adults and children [6]. Hyperthyroidism as a cause of DCM and heart failure is far less common, especially in pediatric patients. Thyroid hormone regulates key structural and regulatory genes in the cardiac myocyte and conduction system, having a direct effect on heart rate and ventricular contractility. Atrial arrhythmias alone can lead to tachycardia induced cardiomyopathy and heart failure. In thyrotoxic states, there is also an increase in circulating blood volume with decreased pulmonary artery compliance and decreased systemic vascular resistance. Decreased pulmonary artery compliance can present as pulmonary hypertension with decreased exercise tolerance; this is a common association in adults with Graves' disease. The increase in circulating volume, enhanced cardiac contractility, and decreased systemic vascular resistance can lead to an increase in cardiac output of up to 300% higher than normal. These physiologic changes pose an increased burden on the cardiovascular system leading to dilated cardiomyopathy and heart failure [7, 8].

The connection between autoimmune diseases is well described, including an association with autoimmune glomerular disease and Graves' disease. PSGN is mechanistically characterized by immune complexes with subsequent complement activation, which is not autoimmune. It is uncertain whether underlying immunologic derangement may have caused both Graves' disease and PSGN in our patient or whether the simultaneous appearance of these distinct disease processes was purely coincidental. Perhaps in the future, this association between GD and PSGN can be further elucidated.

In conclusion, we report a case of a 5-year-old African American male with hyperthyroidism and thyroid storm precipitated by a streptococcal infection complicated by poststreptococcal glomerulonephritis presenting with dilated cardiomyopathy and decreased heart function. This case is unique because of the low incidence of thyroid storm in the pediatric population and the concomitant finding of a rare cardiac and renal disease at presentation. To the authors' knowledge, this association has not been previously reported.

## Figures and Tables

**Table 1:** Trend of thyroid function tests during the patient's hospital course.

Lab value	Day 1 (day of admission)	Day 4	Day 7	Day 9	Day 15 (discharge)
TSH (*μ*U/mL)	<0.006	<0.006	<0.006	<0.006	<0.006
Free T4 (ng/dL)	7.77	3.83	2.42	1.76	1.29
TSI (%)	464%				
Anti-TPO (IU/mL)	1.3 (indeterminate)				
Antithyroglobulin (IU/mL)	1.3 (negative)				
